# Chest tube removal at different gas flows in prolonged air leak: a randomized non-inferiority trial

**DOI:** 10.1093/ejcts/ezae097

**Published:** 2024-03-13

**Authors:** Xinyang Li, Hang Yang, Yongsheng Cai, Xin Ye, Qirui Chen, Ying Ji, Jing Wang, Yili Fu, Bin Hu, Jinbai Miao

**Affiliations:** Department of Thoracic Surgery, Beijing Institute of Respiratory Medicine and Beijing Chao-Yang Hospital, Capital Medical University, Beijing, China; Department of Thoracic Surgery, Beijing Institute of Respiratory Medicine and Beijing Chao-Yang Hospital, Capital Medical University, Beijing, China; Department of Thoracic Surgery, Beijing Institute of Respiratory Medicine and Beijing Chao-Yang Hospital, Capital Medical University, Beijing, China; Department of Thoracic Surgery, Beijing Institute of Respiratory Medicine and Beijing Chao-Yang Hospital, Capital Medical University, Beijing, China; Department of Thoracic Surgery, Beijing Institute of Respiratory Medicine and Beijing Chao-Yang Hospital, Capital Medical University, Beijing, China; Department of Thoracic Surgery, Beijing Institute of Respiratory Medicine and Beijing Chao-Yang Hospital, Capital Medical University, Beijing, China; Department of Thoracic Surgery, Beijing Institute of Respiratory Medicine and Beijing Chao-Yang Hospital, Capital Medical University, Beijing, China; Department of Thoracic Surgery, Beijing Institute of Respiratory Medicine and Beijing Chao-Yang Hospital, Capital Medical University, Beijing, China; Department of Thoracic Surgery, Beijing Institute of Respiratory Medicine and Beijing Chao-Yang Hospital, Capital Medical University, Beijing, China; Department of Thoracic Surgery, Beijing Institute of Respiratory Medicine and Beijing Chao-Yang Hospital, Capital Medical University, Beijing, China

**Keywords:** Prolonged air leak, Chest drainage tube removal, Pulmonary surgery, Digital drainage system, Intrapleural pressure

## Abstract

**OBJECTIVES:**

To evaluate the safety and feasibility of removing drainage tubes at larger size of air leak in patients with prolonged air leak after pulmonary surgery.

**METHODS:**

Ninety-five patients who underwent pulmonary surgery with prolonged air leak in our centre were enrolled in this randomized controlled, single-centre, non-inferiority study. The drainage tube was clamped with a stable size of air leak observed over the last 6 h, which was quantified by gas flow rate using the digital drainage system. The control group (*n* = 48) and the study group (*n* = 46) had their drainage tube clamped at 0–20 ml/min and 60–80 ml/min, respectively. We continuously monitored clinical symptoms, conducted imaging and laboratory examinations, and decided whether to reopen the drainage tube.

**RESULTS:**

The reopening rate in the study group was not lower than that in the control group (2.08% vs 6.52%, *P* > 0.05). The absolute difference in reopening rate was 4.44% (95% confidence interval –0.038 to 0.126), with an upper limit of 12.6% below the non-inferiority margin (15%). There were significant differences in the length of stay [16.5 (13–24.75) vs 13.5 (12–19.25), *P* = 0.017] and the duration of drainage [12 (9.25–18.50) vs 10 (8–12.25), *P* = 0.007] between the control and study groups. No notable differences were observed in chest X-ray results 14 days after discharge or in the readmission rate.

**CONCLUSIONS:**

For patients with prolonged air leak, removing drainage tubes at larger size of air leak demonstrated similar safety compared to smaller size of air leak, and can shorten both length of stay and drainage duration.

**Clinical trial registration number:**

Name of registry: Gas flow threshold for safe removal of chest drainage in patients with alveolar-pleural fistula prolonged air leak after pulmonary surgery. Registration number: ChiCTR2200067120. URL: https://www.chictr.org.cn/

**Figure:**
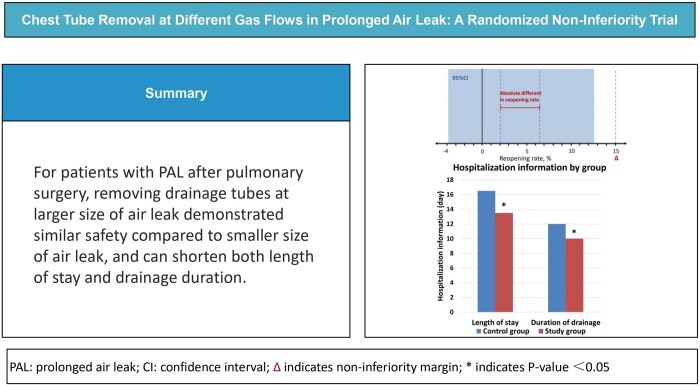

## INTRODUCTION

Thoracic surgery often requires the placement of a chest-closed drainage tube to facilitate the drainage of pleural effusion, improve pneumothorax and promote lung recruitment. The criteria for chest tube removal are primarily based on the volume of fluid drainage and the presence of air leakage. Notably, between 28% and 60% of patients experiencing air leaks achieve complete remission by the 4th postoperative day [1], while the persistence of air leakage over an extended period is categorized as prolonged air leak (PAL), a significant post-pulmonary surgery complication. At present, there is no clear definition of PAL; however, the criteria outlined by the Society of Thoracic Surgeons (STS) and the General Thoracic Surgery Database (GTSD) are commonly referenced, stipulating that air leakage for >5 days post-pulmonary surgery constitutes PAL [2]. This complication is known to occur with an incidence rate of 8–26% [3]. Moreover, the incidence of PAL is associated with surgical procedure, with segmentectomy exhibiting the highest incidence (6.5–14.1%), followed by lobectomy (3.8–8.3%) and wedge resection (3.3%) [4–6]. Intrapleural pressure and the gas flow rate of air leak predict the incidence of PAL after lung surgery [7]. The majority of PAL cases are attributed to alveolar–pleural fistula, primarily resulting from intraoperative lacerations and injuries to the visceral pleura or adjacent lung tissue.

PAL typically follows a self-limiting course but significantly prolongs length of stay (LOS), drainage duration and healthcare expenses for affected patients [8]. Given these challenges, accomplishing early and safe chest tube removal is of clinical importance for patients dealing with PAL after pulmonary surgery. Therefore, we conducted a non-inferiority study to assess the feasibility and safety of chest tube removal at larger size of air leak in comparison to smaller size of air leak, and further explore the relationship between gas flow rate and intrapleural pressure.

## PATIENTS AND METHODS

### Study design and population

This was a prospective randomized controlled, single-centre, non-inferiority study. The study protocol received approval from the Ethics Committee of Affiliated Beijing Chaoyang Hospital of Capital Medical University, Beijing, China in November 2022 (IRB number: 2022-Ke-578). All of the patients participating in this study provided written informed consent to the publication of their study data. Patients who underwent pulmonary surgery between November 2022 and August 2023 at our centre were enrolled.

Inclusion criteria were as follows: (i) underwent pulmonary surgery; (ii) postoperative closed chest drainage; (iii) postoperative air leak >5 days and meeting the Cerfolio classification of air leak greater than or equal to Grade I [9]; and (iv) fluid drainage <200 ml per day on the 5th postoperative day.

Exclusion criteria were as follows: (i) bronchopulmonary fistula diagnosed by bronchoscopy; (ii) abnormal hydrothorax biochemical examination or severe thoracic infection; (iii) NYHA classification III and above or CCS classification III and above; (iv) failure to meet the air leak size criteria of the group after randomization; and (v) lost to follow-up.

Basic information was collected including, gender, age, body mass index, respiratory diseases, hypertension, diabetes mellitus, coronary heart disease, peripheral vascular disease, cerebrovascular disease, tuberculosis, malignant tumour, history of cardiothoracic surgery, neoadjuvant radiochemotherapy, smoking habit, pulmonary function (forced expiratory volume in 1 s/forced vital capacity and carbon monoxide diffusing capacity single-breath method actual/predicted) as well as controlling nutritional status (CONUT) score. Surgical factors included ASA classification, surgical approach, surgical type, upper lobe surgery status, number of lymph node dissections, surgical duration, bleeding volume and presence of pleural adhesions. Hospitalization information included the LOS, duration of drainage, preoperative hospital stays and post-removal of chest tube hospital stay.

### Intervention and procedure

Patients undergoing lung surgery were connected to a water-sealed drainage bottle postoperatively. On the 5th postoperative day, patients were observed for their size of air leak and considered to have PAL if air bubbles overflowed from the water-sealed bottle at normal end-expiration, forced end-expiration, or during a mild or forced cough, and were connected to the digital drainage system (Thopaz, Medela Healthcare, McHenry, IL, USA) with a negative suction pressure of –8 cm H_2_O. All patients were subsequently randomly assigned (1:1) by sequentially numbered, opaque and sealed envelopes to the control group either (0–20 ml/min) or the study group (60–80 ml/min). Another pair of observers conducted the subsequent observations after the drainage tubes were clamped in a double-blind method.

Bronchoscopy was considered to perform in patients with a high suspicion of bronchopulmonary fistula, such as sudden appearance of dyspnoea, hypotension, subcutaneous emphysema, cough with expectoration of purulent fluid, tracheal or mediastinal shift and a reduction or disappearance of pleural effusion.

**Figure 1:**
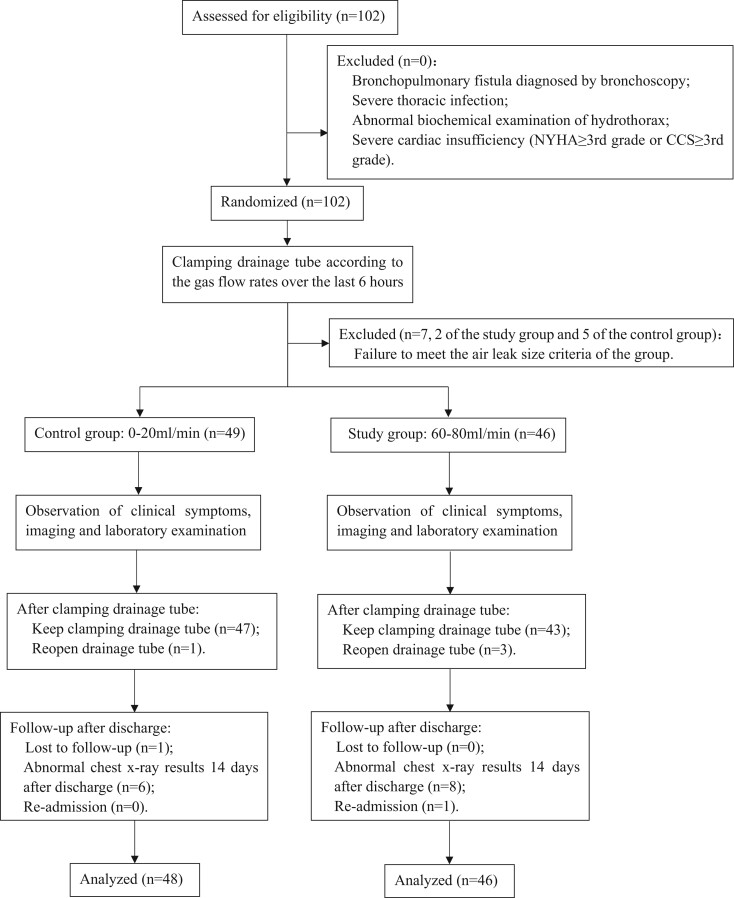
Consolidated Standards of Reporting Trials (CONSORT) flow chart illustrating participants enrolment.

The drainage tube was clamped when the gas flow range stabilized at 0–20 ml/min or 60–80 ml/min for the 2 groups for more than 6 h. Patients were closely monitored for symptoms and oxygen saturation. If corresponding clinical symptoms arose, the drainage tube was reopened, and patients would receive breathing support, run a blood gas analysis and examine chest X-ray (CXR) after stabilization. If the patient had a significantly enlarged pneumothorax comparing with the CXR on the 1st postoperative day or abnormal blood gas analysis, the drainage tube required to be reopened even if there were no obvious clinical symptoms. If patients reported no discomfort, the drainage tube remained clamped for an additional 48 h under observation and removed following no significant changes of CXR, blood gas analysis and biochemical examination. Graphs of gas flow rate and intrapleural pressure before clamping the drainage tube recorded in the digital devices of each participant were exported to read the maximum and minimum of intrapleural pressure. After discharge, all of the participants were instructed to seek medical attention if they experienced uncomfortable symptoms and scheduled for an outpatient visit on the 14th day to re-examine CXR and were followed up via telephone on the 30th and 90th days after discharge. Flow diagram is shown in Fig. 1.

### Study end-points

The primary outcome was categorized as either keeping the drainage tube clamped or reopening the drainage tube. Criteria for reopening the drainage tube included: (i) oxygen saturation: significantly decreased oxygen saturation after ruling out other causes; (ii) clinical symptoms: chest tightness, wheezing, dyspnoea or progressive expansion of subcutaneous emphysema; (iii) chest X-ray results after clamping the drainage tube: enlarged pneumothorax comparing with the CXR on the 1st postoperative day; and (iv) blood gas analysis: hypercapnia, respiratory acidosis or other relevant abnormal results. The secondary outcomes were whether there were abnormalities on CXRs examined on the 14th day after discharge, and readmission within 30 days after discharge.

### Sample size and statistical analysis

Being a non-inferiority study, we computed a sample size of 92 patients, considering a one-sided alpha error of 5% and a power of 80%. The non-inferiority margin could not be calculated using statistical methods due to the absence of unbiased information regarding treatment effects during the trial’s design phase. Based on the data from our centre’s previous study, the incidence of the primary outcome was 2.4% in the control group and 5.1% in the study group. After consideration of the available data and input from experienced clinicians, we arrived at a consensus to establish a 15% absolute difference as the non-inferiority margin, which demonstrated non-inferiority was met and the upper limit of the 95% confidence interval for the incidence of the primary outcome in the control group fell below 15%. Sample size calculation was conducted using PASS 11 based on non-inferiority tests for 2 proportions. After setting the reference group proportion as 2.4% and non-inferiority proportion as 17.4%, the final calculated sample size was 92 patients. Factoring in a potential dropout rate of 10%, the final calculated sample size was determined to be 102 participants, with 51 individuals in each group.

Kolmogorov–Smirnov test was used in evaluating the normality of variances in all the data. Continuous variables that followed a normal distribution were presented as mean and standard deviation ($\bar{x}$±s), while non-normally distributed continuous variables and categorical variables were presented as median and quartiles [M (Q1, Q3)]. Statistical analyses included the use of the Mann–Whitney *U* non-parametric test for non-normally distributed continuous variables or *t*-test for normally distributed continuous variables to compare differences between continuous variables. Categorical variables were analysed using Fisher’s exact test if theoretical frequency (TRC) of more than 20% cells was <5, and otherwise using chi-square test. All statistical analyses were conducted using SPSS 26.0 with a two-sided test, and significance was defined at a *P*-value < 0.05.

## RESULTS

### Comparison of baseline characteristics

A total of 102 patients who underwent pulmonary surgery with postoperative PAL at our centre were recruited into the study between November 2022 and August 2023. The incidence of PAL is 8.3%. Finally, a total of 48 participants were recruited into the control group (2 failed to meet the air leak size criteria and 1 was lost to follow-up after discharge) and 46 participants were recruited into the study group (5 failed to meet the air leak size criteria). The baseline characteristics of both groups are shown in Table 1. The mean age was 58 ± 13 in the control group comparing to 63 ± 11in the study group (*P* > 0.05). Notably, there were no statistically significant differences between the control and study groups in terms of gender, respiratory diseases, hypertension, diabetes mellitus, smoking habit, surgical approach, upper lobe surgery status and presence of pleural adhesions by chi-square test. Moreover, coronary heart disease, peripheral vascular disease, cerebrovascular disease, tuberculosis, malignant tumour, history of cardiothoracic surgery, neoadjuvant radiochemotherapy, ASA classification and surgical type had no statistically differences between the 2 groups by Fisher’s exact test, indicating congruence in the baseline characteristics of the 2 groups.

**Table 1:** Baseline characteristics by group

Variable	Control group (*n* = 48)	Study group (*n* = 46)	*P*-value
Gender			0.817
Male	36 (75.00)	33 (71.74)	
Female	12 (25.00)	13 (28.26)	
Age	58 ± 13	63 ± 11	0.081
BMI	22.11 ± 2.89	21.79 ± 2.56	0.712
Respiratory disease	21 (43.75)	16 (34.78)	0.405
Hypertension	7 (14.58)	13 (28.26)	0.133
Diabetes mellitus	5 (10.42)	11 (23.91)	0.103
Coronary heart disease	3 (6.25)	6 (13.04)	0.311
Peripheral vascular disease	2 (4.17)	5 (10.87)	0.263
Cerebrovascular disease	5 (10.42)	4 (8.70)	>0.99
Tuberculosis	2 (4.17)	2 (4.35)	>0.99
Malignant tumours history	3 (6.25)	6 (13.04)	0.311
Cardiothoracic surgery history	2 (4.17)	3 (6.52)	0.674
Neoadjuvant radiochemotherapy	1 (2.08)	3 (6,52)	0.356
Smoking			0.795
Never	23 (47.92)	22 (47.83)	
Former	8 (16.67)	10 (21.74)	
Present	17 (14.58)	14 (30.43)	
FEV1/FVC	72.63 (66.94–78.66)	71.30 (63.01–77.03)	0.418
<70%	63.89 (49.39–67.02)	61.25 (56.99–66.29)	0.960
≥70%	75.98 (72.48–83.13)	76.54 (72.91–83.08)	0.754
DLCO SB actual/predicted	85.79 ± 21.44	80.10 ± 19.30	0.187
<80%	70.00 (56.40–75.10)	71.60 (56.40–76.80)	0.773
≥80%	94.30 (84.30–104.90)	91.60 (83.30–104.50)	0.946
COUNT score	1 (0.25–2)	1 (0–2)	0.405
≤2	1 (0–2)	1 (0–1)	0.270
>2	3 (3–4)	3 (3–3)	0.255
ASA classification			0.102
I	4 (8.33)	0	
II	30 (62.50)	28 (60.87)	
III	13 (27.08)	18 (39.13)	
IV	1 (2.08)	0	
Surgical approach			0.576
Thoracotomy	9 (18.75)	6 (13.04)	
VATS	39 (81.25)	40 (86.96)	
Surgical type			0.371
Wedge resection	5 (10.42)	5 (10.87)	
Segmentectomy	4 (8.33)	2 (4.35)	
Lobectomy	37 (77.08)	34 (73.91)	
Lobectomy with segmentectomy	1 (2.08)	0	
Bilobectomy	1 (2.08)	5 (10.87)	
Upper lobe surgery	34 (70.83)	26 (56.52)	0.198
Pleural adhesion	23 (47.92)	22 (47.83)	>0.99
Number of lymph node dissection	5 (2.25–7)	6 (3–6.25)	0.662
Surgical duration (min)	150 (120–210)	155 (120–198.75)	0.906
Bleeding volume (ml)	50 (20–137.5)	50 (27.5–100)	0.898

Data are expressed as numbers (%), $\bar{x}$±s or M (Q1–Q3).

BMI: body mass index; DLCO SB: carbon monoxide diffusing capacity single-breath method; FEV1/FVC: forced expiratory volume in 1 s/forced vital capacity; VATS: video-assisted thoracic surgery.

### Comparison of chest tube removal with different gas flow rates

**Figure 2:**
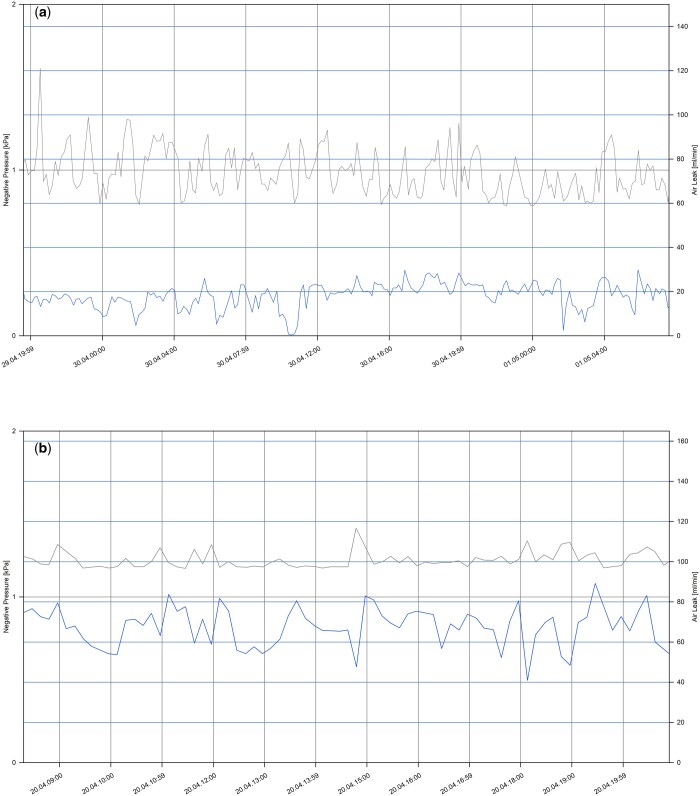
Intrapleural negative pressure and gas flow rate of participants over the last 6 h before clamping the drainage tube. X-axis represent date and time. Grey curves represent intrapleural pressure, and blue curves represent gas flow of air leakage. (**a**) A representative graph from a patient in the control group. (**b**) A representative graph from a patient in the study group.

Gas flow rate graphs over the last 6 h before clamping the drainage tube of both groups are shown in Fig. 2. One patient in the control group and 3 patients in the study group required reopening of the drainage tube during the clamping test due to due to the progressive expansion of subcutaneous emphysema and the onset of chest tightness following the clamping test (Table 2). Importantly, the reopening rates in the control group and the study group were 2.08% and 6.52%, respectively, and the reopening rate in the study group was not lower than that in the control group (*P* > 0.05). The absolute difference in reopening rate was 4.44% (95% confidence interval –0.038 to 0.126), with the upper limit of 12.6% falling below the non-inferiority margin of 15%.

**Table 2:** Post-clamping state of the drainage tube by group

State after clamping the drainage tube	Control group (*n* = 48)	Study group (*n* = 46)	Incidence rate ratio (95% CI)	*P*-value
Keep clamping	47 (97.92)	43 (93.48)		
Reopen	1 (2.08)	3 (6.52)	4.44 (–0.038 to 0.126)	0.613

Data are presented as the number (%) of participants.

CI: confidence interval.

### Comparison of hospitalization information and drainage tube clamping outcomes

There was no significant difference in the preoperative hospital stay and the post-removal of chest tube hospital stay between the 2 groups, a notable difference was observed in the LOS [16.5 (13–24.75) vs 13.5 (12–19.25), *P* = 0.017] and the duration of drainage [12 (9.25–18.50) vs 10 (8–12.25), *P* = 0.007] when comparing the control group to the study group. Notably, the incidence of abnormal CXR results on the 14th day after discharge and the rate of readmission within 30 days did not exhibit significant differences between the 2 groups (Table 3).

**Table 3:** Hospitalization information and drainage tube clamping outcomes by group

Hospitalization information and drainage tube clamping outcomes	Control group (*n* = 48)	Study group (*n* = 46)	*P*-value
Length of stay (day)	16.5 (13–24.75)	13.5 (12–19.25)	0.017
Duration of drainage (day)	12 (9.25–18.50)	10 (8–12.25)	0.007
Preoperative hospital stay (day)	3 (2–5)	3 (2–4.25)	0.815
Post-removal of chest tube hospital stay (day)	1 (0–1)	1 (0–1)	0.788
Chest X-ray on the 14th day			0.571
Normal	42 (87.50)	38 (82.61)	
Abnormal	6 (12.50)	8 (17.39)	
Readmission within 30 days	0	1 (2.17)	0.489

Data are presented as days [M (Q1–Q3)] and the number (%) of participants.

### Correlation of intrapleural pressure with gas flow rate

**Figure 3:**
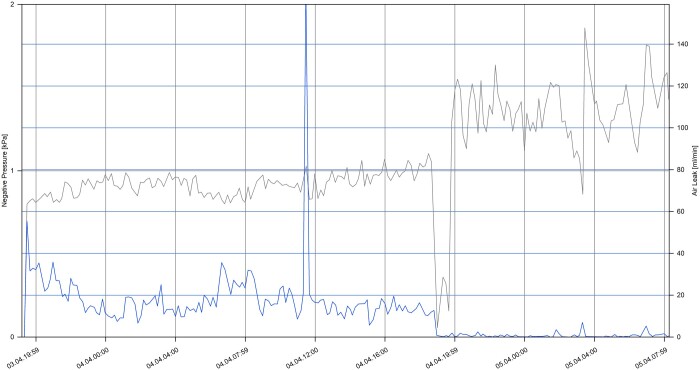
Overall trend of intrapleural negative pressure and gas flow rate of participants before clamping the drainage tube. X-axes represent date and time. Grey curve represents intrapleural pressure, and blue curve represents size of air leak, which is quantified by gas flow rate.

To further explore the relationship between different gas flow rates of air leak and intrapleural pressure in patients with PAL, the overall trend was depicted in Fig. 3, which presented a representative graph of gas flow rate and intrapleural negative pressure. Images of all participants were extracted and the maximum and minimum of intrapleural pressure were read within 6 h prior to clamping test. It is evident that under continuous negative pressure suction (–8 cm H_2_O), as the gas flow rate of air leak decreased, the intrapleural negative pressure increased, and vice versa.

The disparities between the minimum [0.80 (0.75–0.80) vs 0.80 (0.75–0.80), *P* > 0.05] and the maximum [1.40 (1.50–1.60) vs 1.45 (1.30–1.60), *P* > 0.05] of intrapleural negative pressure were not deemed statistically significant after conducting a detailed comparison of the minimum and the maximum of intrapleural pressure within the last 6 h prior to clamping the drainage tube (Table 4). This indicates that under the same sustained negative pressure suction conditions, the range of intrapleural pressures across various gas flow rates did not exhibit notable statistical fluctuations.

**Table 4:** Intrapleural negative pressure by group

Intrapleural negative pressure (kPa)	Control group (*n* = 48)	Study group (*n* = 46)	*P*-value
Minimum	0.80 (0.75–0.80)	0.80 (0.75–0.80)	0.674
Maximum	1.40 (1.50–1.60)	1.45 (1.30–1.60)	0.139

Data are presented as negative pressure [M (Q1–Q3)].

## DISCUSSION

This prospective randomized controlled study confirmed the non-inferiority of chest tube removal at a relatively higher gas flow range (60–80 ml/min) compared to a lower gas flow range (0–20 ml/min) for patients experiencing PAL following pulmonary surgery.

In the majority of PAL cases, the assessment of air leak is crucial in determining the optimal timing for drainage tube removal. To the best of our knowledge, no prior studies have explored chest tube removal criteria using gas flow rate representing size of air leak as a parameter following pulmonary surgery. An expert consensus from 2011, drawing on clinical experiences from various centres, proposed that chest drainage tubes could safely be removed in patients with postoperative air leaks when gas flow rates were below 40 ml/min and remained stable or decreased over the preceding 6–8 h [10]. Some studies have even suggested that gas flow rates lower than 30 ml/min for more than 8 h signify the cessation of air leaks [11]. Previous research at our centre has suggested the potential for chest tube removal at a higher gas flow rate. However, these findings require more robust, evidence-based confirmation. Therefore, we initiated a prospective study to substantiate the feasibility of removing chest drains at higher gas flow rates under stable pressure conditions. This approach to early removal of chest tube holds the potential to reduce duration of drainage and LOS. In addition, interventions such as surgical treatment and pleurodesis are also the commonly performed procedures to overcome PAL to reduce LOS. Autologous blood patch pleurodesis was effective in the treatment of PAL and a Heimlich valve was beneficial for lung expansion in patients whom the air leak stopped or significantly decreased [12]. Chemical pleurodesis with talc, doxycycline and tetracycline has good therapeutic effect on PAL as well [5]. For PALs in patients, there has been a lack of expert consensus regarding best practice.

One of the reasons why patients with postoperative PAL can be extubated early at a high gas flow is the dynamic equilibrium of gas within the pleural cavity. During exhalation, intrapleural pressures exceed the suction pressure, allowing the expulsion of accumulated air during inhalation through the chest tube [13]. A dynamic equilibrium can be established between gas reabsorption from the pleural cavity and the presence of an alveolar–pleural fistula. This allows for partial reabsorption of gas within the pleural cavity. Consequently, patients with minimal pneumothorax can be conservatively managed under close observation [14, 15].

Another important reason for early removal of chest tube at a higher gas flow rate in patients with PAL is stable intrapleural pressure. Analysis of curves of gas flow rate and intrapleural negative pressure revealed that under the same constant negative pressure suction when the gas flow rate was stable within a certain range, the intrapleural pressure was also relatively stable within a certain range, and the range of this relatively stable intrapleural pressure did not change significantly under different gas flow rates. Furthermore, Brunelli *et al.* have shown that a higher gas flow rate and a higher pressure differential were associated with a higher incidence of PAL [7], indicating that, along with gas flow rate, intrapleural pressure also plays a crucial role in air leak occurrence. In addition, our results indicated that with a stable gas flow range of 0–20 ml/min or 60–80 ml/min, the intrapleural pressure can be maintained in the same relatively stable range independent of the flow rate, and this may explain the feasibility of chest tube removal at certain gas flow rate.

The possibility of safely removing the drainage tube at a specific gas flow rate is strongly associated with the type of air leak as determined by intrapleural pressure. Air leaks can be categorized as pressure-dependent and pressure-independent by performing a clamping test. In patients with pressure-dependent air leaks, intrapleural pressure remains relatively stable during the clamping test. During this test, the drainage tube is clamped for 20–30 min, with continuous monitoring of intrapleural pressure. A stable or slightly fluctuating end-expiratory intrapleural pressure during the clamping test indicates a pressure-dependent air leak, while a sustained increase in end-expiratory intrapleural pressure without a significant plateau signifies a pressure-independent air leak [16]. A significant 80% of PAL following pulmonary surgery are attributed to pressure-dependent air leaks [17], often caused by surgical injuries leading to lung tissue wounds or a mismatch between the residual lung and the pleural cavity.

In this study, 1 participant who passed the clamping test and had the drainage tube removed successfully was excluded due to loss to follow-up after discharge, although this might lead to an overestimation of the reopening rate in the control group, the same results can be still obtained. Four cases required the reopening of the drainage tube due to the progressive expansion of subcutaneous emphysema and the development of wheezing symptoms following the clamping test. This suggested the possibility of pressure-independent air leak. However, majority of patients were removed the drainage tube successfully. Observation of the intrapleural negative pressure curves before clamping the drainage tube revealed that when the gas flow rate stabilized within a certain range, the intrapleural pressure tended to stabilize, and fluctuations in the gas flow rate did not cause significant change in the intrapleural pressure.

These results are also similar to those reported in previous studies. Chopra *et al.* reported a case where a patient underwent right middle lobectomy for lung malignancy and developed dyspnoea 3 weeks post-surgery, with a chest X-ray revealing pneumothorax. Continuous air leakage persisted for 2 weeks after thoracentesis and chest drainage tube placement. Subsequently, the drainage tube was removed after a clamping test, and intrapleural pressure manometry confirmed a pressure-dependent air leak. The patient was followed up for a year after discharge and repeated chest X-ray examinations showed stable localized pneumothorax [18]. Walker *et al.* suggested that pressure-dependent air leaks with continuous chest drainage may be exacerbated by an increased pressure gradient between the site of air leak in lung tissue and the pleural cavity [19]. Continuous monitoring of intrapleural pressure after clamping the drainage tube is thus imperative, and in cases where the air leak is confirmed as pressure-dependent and the patient remains asymptomatic, the drainage can be expeditiously removed following imaging evaluation [16]. Therefore, for patients experiencing PAL after pulmonary surgery, continuous monitoring of intrapleural pressure after drainage tube clamping holds equal clinical significance to the continuous monitoring of gas flow.

This study was the 1st to explore the chest tube removal threshold of gas flow in patients with PAL after pulmonary surgery, providing initial evidence for the safety and feasibility of chest tube removal at a relatively higher gas flow rate. Simultaneously, early chest tube removal can significantly reduce LOS, drainage duration and postoperative pain caused by drainage tube stimulation. However, this study has some limitations: (i) the sample size was relatively small, which potentially leads to higher variability and results in lack of power. (ii) Short-term focus of this study limited the ability to conclusively determine the long-term benefits or risks associated with chest tube removal at higher gas flow rates. (iii) This study being conducted at a single centre introduces bias. Further multicentre validation is required. Further research should investigate the relationship between leak type, including pressure-dependent and pressure-independent air leaks, and gas flow rate in patients with PAL after pulmonary surgery.

## Data Availability

The data underlying this article will be shared on reasonable request to the corresponding author.

## ABBREVIATIONS

CONUT: Controlling nutritional status
GTSD: General Thoracic Surgery Database
LOS: length of stay
PAL: Prolonged air leak
STS: Society of Thoracic Surgeons

## References

[1] Mueller MR , Marzluf BA. The anticipation and management of air leaks and residual spaces post lung resection. J Thorac Dis 2014;6:271–84. 24624291 10.3978/j.issn.2072-1439.2013.11.29 PMC3949188

[2] Clark JM , Cooke DT, Brown LM. Management of complications after lung resection: prolonged air leak and bronchopleural fistula. Thorac Surg Clin 2020;30:347–58. 32593367 10.1016/j.thorsurg.2020.04.008 PMC10846534

[3] Singhal S , Ferraris VA, Bridges CR, Clough ER, Mitchell JD, Fernando HC et al Management of alveolar air leaks after pulmonary resection. Ann Thorac Surg 2010;89:1327–35. 20338378 10.1016/j.athoracsur.2009.09.020

[4] Suzuki K , Saji H, Aokage K, Watanabe S-I, Okada M, Mizusawa J et al; Japan Clinical Oncology Group. Comparison of pulmonary segmentectomy and lobectomy: safety results of a randomized trial. J Thorac Cardiovasc Surg 2019;158:895–907. 31078312 10.1016/j.jtcvs.2019.03.090

[5] Dugan KC , Laxmanan B, Murgu S, Hogarth DK. Management of persistent air leaks. Chest 2017;152:417–23. 28267436 10.1016/j.chest.2017.02.020 PMC6026238

[6] Gonzalez M , Karenovics W, Bédat B, Forster C, Sauvain M-O, Triponez F et al Performance of prolonged air leak scoring systems in patients undergoing video-assisted thoracoscopic surgery segmentectomy. Eur J Cardiothorac Surg 2022;62. 10.1093/ejcts/ezac100 35229873

[7] Brunelli A , Cassivi SD, Salati M, Fibla J, Pompili C, Halgren LA et al Digital measurements of air leak flow and intrapleural pressures in the immediate postoperative period predict risk of prolonged air leak after pulmonary lobectomy. Eur J Cardiothorac Surg 2011;39:584–8. 20801054 10.1016/j.ejcts.2010.07.025

[8] Yoo A , Ghosh SK, Danker W, Kassis E, Kalsekar I. Burden of air leak complications in thoracic surgery estimated using a national hospital billing database. Clinicoecon Outcomes Res 2017;9:373–83. 28721079 10.2147/CEOR.S133830 PMC5498775

[9] Kida H , Muraoka H, Morikawa K, Inoue T, Mineshita M. Pleurodesis after bronchial occlusion for inoperable secondary spontaneous pneumothorax. J Bronchology Interv Pulmonol 2021;28:290–5. 34191760 10.1097/LBR.0000000000000785

[10] Brunelli A , Beretta E, Cassivi SD, Cerfolio RJ, Detterbeck F, Kiefer T et al Consensus definitions to promote an evidence-based approach to management of the pleural space. A collaborative proposal by ESTS, AATS, STS, and GTSC. Eur J Cardiothorac Surg 2011;40:291–7. 21757129 10.1016/j.ejcts.2011.05.020

[11] Pompili C , Detterbeck F, Papagiannopoulos K, Sihoe A, Vachlas K, Maxfield MW et al Multicenter international randomized comparison of objective and subjective outcomes between electronic and traditional chest drainage systems. Ann Thorac Surg 2014;98:490. 24906602 10.1016/j.athoracsur.2014.03.043

[12] Apilioğulları B , Dumanlı A, Ceran S. Application of autologous blood patch in patients with non-expanded lungs and persistent air leak. Turk Gogus Kalp Damar Cerrahisi Derg 2020;28:521–6. 32953216 10.5606/tgkdc.dergisi.2020.18983 PMC7493595

[13] Mentzer SJ , Tsuda A, Loring SH. Pleural mechanics and the pathophysiology of air leaks. J Thorac Cardiovasc Surg 2018;155:2182–9. 29397977 10.1016/j.jtcvs.2017.12.062 PMC7263434

[14] Paskaradevan J , Sayad E, Sockrider M. What is a spontaneous pneumothorax? Am J Respir Crit Care Med 2020;202:P33–4. 33320071 10.1164/rccm.20212P33

[15] Luh SP. Review: diagnosis and treatment of primary spontaneous pneumothorax. J Zhejiang Univ Sci B 2010;11:735–44. 20872980 10.1631/jzus.B1000131 PMC2950234

[16] Chopra A , Doelken P, Hu K, Huggins JT, Judson MA. Pressure-dependent pneumothorax and air leak—physiology and clinical implications. Chest 2023;164:796–805. 37187435 10.1016/j.chest.2023.04.049

[17] Chopra A , Hu K, Judson MA, Fabian T, Nabagiez JP, Feustel PJ et al Association between drainage-dependent prolonged air leak after partial lung resection and clinical outcomes: a prospective cohort study. Ann Am Thorac Soc 2022;19:389–98. 34715010 10.1513/AnnalsATS.202103-235OC

[18] Chopra A , Doelken P, Judson MA, Huggins T. The pressure-dependent air leak after partial lung resection. Thorax 2017;72:290–1. 27672119 10.1136/thoraxjnl-2016-208884

[19] Walker SP , Hallifax R, Rahman NM, Maskell NA. Challenging the paradigm of persistent air leak: are we prolonging the problem? Am J Respir Crit Care Med 2022;206:145–9. 35353640 10.1164/rccm.202109-2149PP

